# Snakes Represent Emotionally Salient Stimuli That May Evoke Both Fear and Disgust

**DOI:** 10.3389/fpsyg.2019.01085

**Published:** 2019-05-09

**Authors:** S. Rádlová, M. Janovcová, K. Sedláčková, J. Polák, D. Nácar, Š. Peléšková, D. Frynta, E. Landová

**Affiliations:** ^1^National Institute of Mental Health, Klecany, Czechia; ^2^Department of Zoology, Faculty of Science, Charles University, Prague, Czechia; ^3^Department of Psychology, Faculty of Arts, Charles University, Prague, Czechia

**Keywords:** snakes, fear, disgust, affective stimuli, self-reported emotion, emotional response

## Abstract

Humans perceive snakes as threatening stimuli, resulting in fast emotional and behavioral responses. However, snake species differ in their true level of danger and are highly variable in appearance despite the uniform legless form. Different snakes may evoke fear or disgust in humans, or even both emotions simultaneously. We designed three-step-selection experiments to identify prototypical snake species evoking exclusively fear or disgust. First, two independent groups of respondents evaluated 45 images covering most of the natural variability of snakes and rated responses to either perceived fear (*n* = 175) or disgust (*n* = 167). Snakes rated as the most fear-evoking were from the family Viperidae (*Crotalinae*, *Viperinae*, and *Azemiopinae*), while the ones rated as the most disgusting were from the group of blind snakes called Typhlopoidea (*Xenotyphlopinae*, *Typhlopinae*, and *Anomalepidinae*). We then identified the specific traits contributing to the perception of fear (large body size, expressive scales with contrasting patterns, and bright coloration) and disgust (thin body, smooth texture, small eyes, and dull coloration). Second, to create stimuli evoking a discrete emotional response, we developed a picture set consisting of 40 snakes with exclusively fear-eliciting and 40 snakes with disgust-eliciting features. Another set of respondents (*n* = 172) sorted the set, once according to perceived fear and the second time according to perceived disgust. The results showed that the fear-evoking and disgust-evoking snakes fit mainly into their respective groups. Third, we randomly selected 20 species (10 fear-evoking and 10 disgust-evoking) out of the previous set and had them professionally illustrated. A new set of subjects (*n* = 104) sorted these snakes and confirmed that the illustrated snakes evoked the same discrete emotions as their photographic counterparts. These illustrations are included in the study and may be freely used as a standardized assessment tool when investigating the role of fear and disgust in human emotional response to snakes.

## Introduction

Due to a long co-evolutionary history with snakes, both humans and non-human primates evolved specific neural mechanisms for rapid snake recognition (Isbell, 2006; LoBue and DeLoache, 2008; Öhman et al., 2012; Van Le et al., 2013; Baynes-Rock, 2017). Among evolutionarily irrelevant (neutral) stimuli, snake pictures act as strong distractors (Soares et al., 2009a) and are detected faster (LoBue and Deloache, 2011; Soares et al., 2014) than, for example, flowers and mushrooms, but not faster than stimuli of modern threats such as guns (Fox et al., 2007; Zsido et al., 2018b). Moreover, EEG studies show that neural processing of snake stimuli is prioritized when compared to other animals such as spiders and birds (van Strien et al., 2014).

LoBue and Deloache (2011) provide evidence that it is the distinctive coiled snake morphology that attracts prioritized human attention. However, recent research has shown that the pattern of snake scales is also important. The human brain reacts much faster to pictures of snake skin than similarly, colored bird feathers (van Strien and Isbell, 2017). Moreover, some naturally occurring shapes and patterns are perceived negatively, and processed faster than other patterns, such as sharp edges (Guthrie and Wiener, 1966; Bar and Neta, 2006, 2007), zig zag patterns (Üher, 1991), or strong contrasting patterns in general (Näsänen et al., 2001). Souchet and Aubret (2016) proposed that venomous snakes adopt this phenomenon and use contrasting patterns and morphology with sharp edges as an aposematic signal to deter enemies and communicate their dangerousness. The ability to recognize danger presented by a snake by only a pictured skin detail was also observed in Vervet monkeys (Isbell and Etting, 2017), and Capuchin monkeys were able to recognize whether the presented snake was dangerous or harmless only based on the skin pattern (Meno et al., 2013). On the other hand, Prokop et al. (2018) showed that aposematic coloration did not play a crucial role in eliciting high fear of snakes as both black-and-white and color images of aposematic and cryptic snakes evoked a similar level of increased fear.

Both the typical snake body shape and color pattern seem to be important elicitors of neural mechanisms for rapid snake detection, although it remains unclear what is the typical snake pattern that elicits these reactions. Nowadays, as much as 3 709 snake species from 25 families (Uetz et al., 2018) are described. Many of them differ in ecology, size, skin pattern, behavior, and other aspects. Similarly, snakes vary as to the dangerousness they present to humans, including the efficiency of venom and its delivery system, the snake’s size, aggressiveness, and the probability of human encounters. Deadly venomous species can be found within all snake types, i.e., ground, arboreal, fossorial, and aquatic. However, the highest risk is related to two types of snakes (Kasturiratne et al., 2008). First, the venomous vipers or viper-like species are passive predators that use an ambush strategy for hunting. Their dangerousness lies in the unexpected risk, such as the possibility to step on the snakes by mistake (though some species actively warn potential enemies using demonstrative attacks or various acoustic signals such as hissing or rattling). The second type of snakes posing a high risk for humans are the elapids. No less venomous than the vipers, these active predators are much more mobile. They also rely on acoustic or visual signals (e.g., the typical cobra stance) as well as their high speed, which allows them to actively avoid an unwanted confrontation with humans or other enemies (Valenta, 2008). In Africa, the continent of human origin (Grine et al., 2009), the highest number of death by snakes is caused by vipers (Trape et al., 2001), especially the West African carpet viper (*Echis ocellatus*) and Gaboon viper (*Bitis gabonica*; Chippaux, 1998). Each of these snakes is characterized by specific morphological traits that can be easily recognized, e.g., the viperids possess a triangular-shaped head and sharp, visually discrete scales. Many deadly snakes have contrasting (aposematic) skin coloration patterns, by which they make themselves clearly visible. It is possible that humans do not perceive all snake species as one, generally threatening stimulus, but rather distinguish among the snakes that are deadly and react appropriately to the actual threat.

Most studies focusing on snake fear ignore the enormous variability of snakes. However, little is known about the actual effect of particular morphology of snakes on human emotional reactions. Traditional assumptions in psychology research is that the primary emotion involved in ophidiophobia is fear (Öhman and Mineka, 2001, 2003; Soares et al., 2009b), however, our recent studies show that disgust is involved in emotional evaluation of snakes and other vertebrates as well (Polák et al., submitted; Frynta et al., unpublished; see also Prokop and Fančovičová, 2013). Similarly Davey (1994) has shown that snakes are rated as fear-evoking as a result of elevated disgust sensitivity levels, and it is possible that some snake morphotypes may elicit a primary emotion of disgust. Disgust is also of prior interest to clinical researches as increased propensity (i.e., individual tendency to experience disgust) and sensitivity to disgust (i.e., negative appreciation of experiencing disgust) has been demonstrated to play a significant role in etiology of various psychological disorders ranging from specific animal phobia (e.g., arachnophobia: de Jong and Merckelbach, 1998; Woody et al., 2005; Muris et al., 2008; Olatunji et al., 2011) and blood-injection-injury phobia (Sawchuk et al., 2000; Cisler et al., 2009b). Therefore, it may also be involved in the onset and maintenance of snake phobia (Klieger and Siejak, 1997).

Both fear and disgust are considered to be basic emotions, with a universal distinctive facial expression and physiological response among humans and non-human primates (Ekman, 1992). Furthermore, some authors argue that fear and disgust could have opposing effects on sensory perception and attention (Buck et al., 2018). From a biological perspective, the two emotions are similar, as their purpose is to induce an adaptive reaction to life-threatening stimuli, increasing the chances of survival. However, fear arises in cases of immediate threat, which appears suddenly, unexpectedly, and presents a direct risk of injury or death. The resulting physiological reaction leads to activation of the sympathetic nervous system and a cascade of events, including rapid heart and respiratory rates, increased blood pressure, dilated pupils, muscle contraction, and increased perspiration (Barrett et al., 2016). In contrast, disgust is a reaction to stimuli perceived as potential sources of contamination or pathogens (Curtis et al., 2004; Curtis, 2011). The threat is not imminent, but rather takes effect after a prolonged period of time. The physiological reaction to a disgusting stimulus is more variable. Compared with fear, the parasympathetic pathways are activated (de Jong et al., 2011), leading to either an increase or decrease in skin conductance and blood pressure (Stark et al., 2005) and reduced heart rate and respiration (Gilchrist et al., 2016). The reason for this may be the fact that disgust is much more variable emotion than fear; thanks to this pre-adaptation, it evolved into a number of other functional circuits, fundamentally different from its original purpose. Disgust is thus linked to various other triggers such as violation of social norms, political beliefs, sexual orientation, and people from other social groups, etc. (Rozin et al., 1999).

The elicitors of fear and disgust also differ in many aspects. Specific characteristics of an animal can trigger specific emotions, and these characteristics can differ even within an animal group otherwise positively evaluated by humans. A good example is frogs and other amphibians, a vertebrate class which generally is thought to elicit disgust (Angyal, 1941; Davey, 1994; Tomažič, 2011; Prokop et al., 2016). Among them, frogs are listed as one of the animals that are very often objects of specific phobias associated with strong disgust feelings (Doctor et al., 2010). However, only a few frog species with specific characteristics are ranked as disgusting. These characteristics include a round, chunky body shape, small eyes, warts, drab colors, pink color, and white light reflection that gives the impression of a slimy object (Frynta et al., unpublished). Moreover, even mammals, generally a very popular and well-perceived group of animals, contain species with characteristics that make them look dangerous or disgusting. For example, underground-dwellers of a small body size, round shape, and reduced eyes, e.g., mole rats (Bathyergidae), marsupial moles (Notoryctidae), and moles (Talpidae) are often seen as ugly or disgusting (Landová et al., 2018b; see also Frynta et al., 2013 for the rankings of “beauty” and “ugliness”). In contrast, mammals who are thought to be the most dangerous and capable of evoking the highest fear are generally of large body size, such as big cats (although these can also elicit positive emotions at the same time; Landová et al., 2018b; see also de Pinho et al., 2014).

Furthermore, when an animal has a combination of characteristics, the animal can elicit mixed emotions. A snake can be rated as beautiful (Marešová et al., 2009a,b; Frynta et al., 2011; Landová et al., 2012), i.e., eliciting positive aesthetic emotion (Schindler et al., 2017), and fear-eliciting at the same time (Landová et al., 2012, 2018a). It is easy to imagine that a mix of negative emotions, i.e., fear and disgust, could be elicited at the same time as well. Thus, to study emotions triggered by snakes correctly, one must choose the right experimental stimuli and mind the differences between various morphotypes.

When studying human-perceived emotions elicited by snakes, one must not forget that respondents with different susceptibility to snake fear or general disgust and/or anxiety may answer differently. A great amount of literature exists that describes differences in behavior of snake-fearful or phobic respondents in comparison to controls (see, e.g., Öhman and Soares, 1994; Öhman et al., 2001; Schaefer et al., 2014). Given all these differences between phobics and healthy controls, one might also expect variation between these groups in fear and disgust evaluation of snake pictures. Therefore, it is necessary to first test the responses of healthy subjects to fear-eliciting and disgust-eliciting snake images and subsequently administer the same images to snake phobics for a comparison. The participation of each emotion and the relationship between them is crucial when we try to better understand the different line of evidence for involvement of these emotions in causing specific animal phobias (Woody and Teachman, 2000; Cisler et al., 2009a).

There is a lot of picture databases with known evaluations of self-reported emotional responses (usually valence, arousal, and dominance, supplemented with emotional categories that include fear and disgust), e.g., IAPS (Lang et al., 1988; Mikels et al., 2005; Libkuman et al., 2007), NAPS (Marchewka et al., 2014; Riegel et al., 2016), SFIP (Michałowski et al., 2017), DIRTI (Haberkamp et al., 2017), or GAPED (Dan-Glauser and Scherer, 2011). However, these databases consist of pictures with very varying properties including background, colors, lightness, focus on detail, etc. The last mentioned database even includes a separate category of snake pictures, but again the pictures portray the animals in very different contexts and environments, including human hands grabbing the snakes. Moreover, the snakes lack taxonomic description and species identification (e.g., the GAPED database even includes one glass lizard among the snakes). And although these pictures may evoke the particular emotions, it is not known which part of the picture is the main elicitor – is it the snake itself, its particular appearance/position, or something else such as its interaction with the background? It is well known that even basic characteristics of pictures such as the color, luminance, and shape structure affect human evaluation of emotion or preference (Üher, 1991; Bar and Neta, 2006; Silvia and Barona, 2009; Lišková and Frynta, 2013; Lišková et al., 2015; Ptáčková et al., 2017; Landová et al., 2018b; Rádlová et al., 2018). Moreover, such stimuli may be suitable for usage in experiments measuring self-reported or physiological response to a single picture, but are inappropriate for studies requiring block-type design (such as fMRI or EEG), because the stimuli are too variable and could elicit different reactions within a block.

Without a precise selection of well-characterized and standardized stimuli, further studies focused on the variability among respondents may result in misinterpretations. Thus, the main goal of this study was to examine the pattern of variability among various snake stimuli and to develop one homogenous set eliciting fear and another homogenous set eliciting disgust. We are very aware that individual differences in snake fear and general disgust propensity may influence the evaluation of snake species. To examine the differences among the respondents in terms of snake-elicited fear and disgust, we collected additional data about the participants including questionnaires such as the SNAQ (Polák et al., 2016, see also Zsido et al., 2018a for its shorter version) and a self-reported response based on a method more suitable for this kind of experiment. However, due to length limitation, these data will be published in a separate article (in prep.) as this paper is mainly focused on the differences among the picture stimuli. In other words, in this paper, we aim to study the effect of different stimuli on the respondents and to create categories of snake stimuli based on exclusive reactions.

More precisely, the general aims of this study were to (1) examine the full morphological variability of snakes in terms of human emotional responsiveness and to analyse the specific characteristics contributing to the human perception of fear and/or disgust. Then, based on the results, to (2) create two sets of visual snake stimuli that would evoke exclusively fear or disgust in human respondents. Finally, we aimed to (3) confirm the results of Experiment 2 on a reduced set of illustrated stimuli that could be make freely available for download and for usage in experimental studies of human-perceived emotions elicited by snakes.

## Experiment 1. Full Morphological Variability of Snakes

### Materials and Methods

#### Selection and Preparation of Stimuli

In case of snakes, subfamilies represent the optimal taxonomical unit that reflect the most distinctive differences between the groups. Thus, we randomly selected one species from each of the extant snake subfamilies (45 in total; see Pyron et al., 2013), inhabiting various parts of the world. To avoid over-representation of a particular genera, we randomly selected a genus and then one of its species.

**FIGURE 1:**
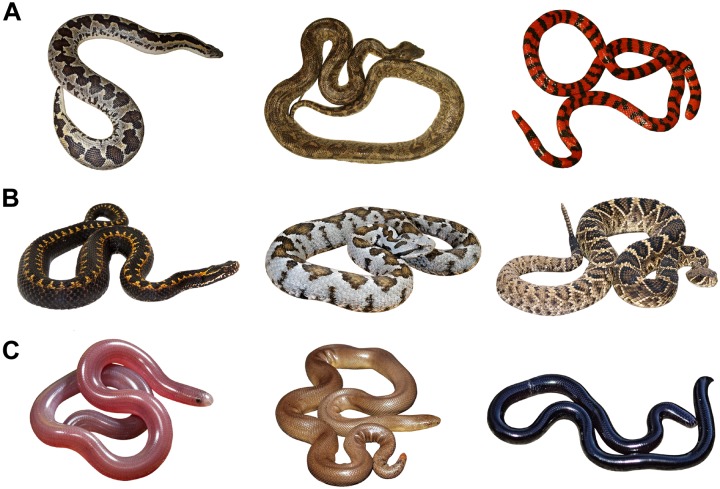
Examples of picture stimuli used in Experiments 1 **(A)** and 2 **(B,C)**. The snake species are as follows, from left to right: **(A)** Rough-tailed Sand Boa (*Eryx conicus*) and Madagascar Tree Boa (*Sanzinia madagascariensis*), both photos by O. Šimková, used with a permission; Coral Cylinder Snake (*Anilius scytale*), photo by Eduardo Santos via Wikimedia Commons, modified. **(B)** Fear-eliciting snakes: Orlov’s Viper (*Vipera orlovi*), photo by Venom Tales via Flickr, modified; Ottoman Viper (*Montivipera xanthina*), photo by Benny Trapp via Wikimedia Commons, modified; Eastern Diamondback Rattlesnake (*Crotalus adamanteus*), photo by Pierson Hill, used with a permission. **(C)** Disgust-eliciting snakes: Southern Blind Snake (*Anilios australis*), photo by Stephen Zozaya, used with a permission; Northern Rubber Boa (*C. bottae*), photo by Gary Nafis, used with a permission; Brahminy Blind Snake (*Indotyphlops braminus*), photo by Alexandre Roux via Flickr, modified.

For each of the selected species, we searched a representative picture of an adult individual on the Internet. It has been shown in our previous study that relative fear rankings of photos and live snakes highly correlate (*r* = 0.78), thus using picture stimuli may provide valid results regarding human responses to snakes (Landová et al., 2012). Our search criteria included photos that were of a good quality and publicly available, i.e., licensed under the Creative Commons license or a written permission was given to us by the authors (For a full list of included pictures, see Supplementary Material 1). After collecting the pictures, we modified them to create a standardized setting (using GIMP 2.8, Kimball et al., 1995): we placed the snakes on a unified white background, scaled and rotated them to similar relative size and position with each snake facing the same direction (see Figure 1). The total of 45 picture stimuli (one for each of the selected species) was printed on matte 10 × 15 cm cards.

#### Testing Emotional Response to Snakes

To test the emotional response to snakes by human respondents, we adopted a widely used method of sorting picture stimuli according to a given scale (e.g., Marešová et al., 2009a,b; Lišková et al., 2015; etc.). The reason for which we chose this method is because the main aim of this paper is to examine the variability among the snake species in terms of human-perceived fear and disgust. In other words, we ask whether different snake species/morphotypes affect the respondents differently. For this kind of study, the sorting method is optimal as it maximizes the differences among species. However, as the acquired rankings for each stimulus are only relative, this method is not a good choice for studies focused on the variability among the respondents (e.g., whether snake-fearful respondents rank the snakes differently). For such kind of study, a different ranking method would be more suitable, such as the VAS, Borg, or Likert-type scale (Likert, 1932; Grant et al., 1999). Note that we also collected this kind of data, the results of which will be published in a separate article.

All respondents (*n* = 342) were Czechia residents (aged 19 – 67; mean age = 28.18; *SD* = 9.29). Sample included students and other respondents that volunteered based on informational pamphlets. Other volunteers were recruited using the snowball sampling method (Goodman, 1961; Biernacki and Waldorf, 1981). Our aim was to keep the ratio of respondents with biological and humanities education balanced.

First, each of the respondents filled a personal questionnaire about their age and gender, and signed an informed consent. In addition, each respondent provided us with a rank of their self-perceived affiliation toward snakes on a seven-point Likert scale (1 = I like snakes very much, I would like to keep one as a pet; 4 = neutral affiliation; and 7 = I dislike/hate snakes very much, I fear them; Wuensch, 2015). This variable is further referred to as “affiliation.”

Then, we distributed all 45 snake cards on a well-lit table in a random order. The respondents were asked to imagine the depicted snakes as real animals. Their task was to pick up the picture of a snake that was most fear-evoking, then to pick up the second most fear-evoking snake, until they picked up the least fear-evoking snake on the table. In total, 97 women and 78 men sorted the picture set according to perceived fear. Additional 167 respondents (94 women, 73 men) sorted the same picture set according to perceived disgust. Then, because the highest rank obtained this way (i.e., the 45th snake) corresponded to the lowest emotional response, we multiplied the ranks by -1 to obtain more intuitive results.

#### Explanatory Variables

To analyze the effect of morphometric characteristics of the snake species on the human responses, we measured the following variables on the snake photos using the ImageJ 1.40 g (Rasband, 1997): total body length, body width (also referred to as body size), head size (length from the tip of the nose to the end of the jaw), eyes size, neck size (in the thinnest part), and tail width (width of the tail base next to the cloaca). The number of pixels were transformed to centimeters relative to the picture size printed on a 15 × 10 cm card.

To examine the effect of colors on the respondents’ ranking, we used Barvocuc (Rádlová et al., 2016) to extract specific information about hues, lightness, and saturation of each of the stimulus pictures converted to the HSL colorspace. For a detailed description of the Barvocuc software, see Lišková and Frynta (2013) and Lišková et al. (2015). Colors were pre-defined to visually correspond to the snake pictures as accurately as possible: red < 330°; 15°), orange (corresponding to brown in all of the snakes) < 15°; 37°), yellow < 37°; 60°), green < 60°; 200°), blue < 200°; 240°), and violet-rose < 240°; 330°). Further, the red color was divided into two colors as in some snakes it corresponded to reddish brown, while in others, it corresponded to bright red. Three achromatic colors were defined on the basis of the saturation (S) and lightness (L) values, which covered the interval 0–1: black (*L* < 0.27), white (*L* > 0.8), and gray (*S* < 0.1). Mean S and L were also included as explanatory variables. The white background of the stimuli was set to transparent and was excluded from the calculation.

Additionally, we included two more variables extracted from Barvocuc: the pattern, computed using the edge detection method (Sobel, 1978), and opaque pixels, which is the sum of all non-transparent pixels. The latter variable also corresponds to the overall robustness of the depicted snake species. In order to improve normality, the portion of colors, mean S, pattern, and opaque pixels were square-root arcsine transformed prior to the analysis.

#### Statistical Analysis

To quantify and test the congruence in species ranking provided by different respondents, we adopted the Kendall’s Coefficient of Concordance. Significance of differences in mean rank among species was calculated by *post hoc* Friedman–Neményi test as implemented in R-package PMCMR. Prior to analyses, the raw ranks were transformed as follows: each value minus 1 was divided by the number of evaluated species minus 1 (44) and square-root arcsine transformed to achieve a normal distribution. A Principal Component Analysis (PCA) was performed to visualize the multivariate structure of the data sets. A Mann–Whitney *U* test was used as a non-parametric alternative for variables deviating from normality (raw sores). MANCOVA was applied to test the effects of independent explanatory variables. Contribution of the explanatory variables (constrains) to the rankings of the snakes was examined in the Redundancy Analysis (RDA) as implemented in the R package vegan (Oksanen et al., 2017). RDA is a multivariate direct gradient method. It extracts and summarizes the variation in a set of response variables (subjective evaluation of fear and disgust evoked by snakes) that can be explained by a set of explanatory variables. This analysis permits to plot both response and explanatory variables to a space defined by the extracted gradients and enables detection of redundancy (i.e., shared variability) between sets of response and explanatory variables. Statistical significance of the gradients was confirmed by permutation tests. Calculations were performed in R Development Core Team (2010) and Statistica 9.1 (StatSoft Inc., 2010).

### Results

#### Agreement Among Respondents

The overall agreement among respondents on the snake fear (Kendall’s Coefficients of Concordance *W* = 0.254) and disgust rankings (*W* = 0.293) was somewhat low. To correct for potential incongruence caused by an unequal effect of snakes that were ranked as the top, we retested the responses after we divided the set into two halves, i.e., the first half containing 11 snakes rated as the least and 11 as the most fear/disgust-evoking (mean values), and the other half containing 23 snakes in the middle results showed a slight improvement in the agreement on the position of these species (fear-scale extremes *W* = 0.332, middle *W* = 0.091; disgust-scale extremes *W* = 0.409, middle *W* = 0.119). *Post hoc* tests revealed high proportion of significant comparisons among mean ranks of examined stimuli (see Supplementary Material 2a). This confirmed that the congruence among respondents was high enough to extract a reliable order of stimuli according to the examined emotions.

RDA analysis of the fear ranks, which included gender, age, and affiliation toward snakes, revealed that these variables explained only 3.1% of the full variability. Sequential “Type I” ANOVA (*n* permutations = 10 000) confirmed that only the effect of affiliation was significant: affiliation: *F*_1,171_ = 2.272, *p* = 0.0188; gender: *F*_1,171_ = 1.728, *p* = 0.0724; and age: *F*_1,171_ = 1.461, *p* = 0.1315). A reduced model, which included only the affiliation, explained 1.29% of the full variability (ANOVA: *F*_1,173_ = 2.257, *p* = 0.0214). Similar results were found in case of the disgust ranks, although the full model explained more variability (7.57%): affiliation: *F*_1,163_ = 10.715, *p* < 0.0001; gender: *F*_1,163_ = 1.2665, *p* = 0.2091; age: *F*_1,163_ = 1.3744, *p* = 0.1553. Reduced model explained 6.08% variability; ANOVA affiliation: *F*_1,165_ = 10.673, *p* < 0.0001. Since there was no significant effect of age nor gender, the data from both genders were pooled for all further analysis.

#### Factors Determining Fear and Disgust Rankings

**FIGURE 2:**
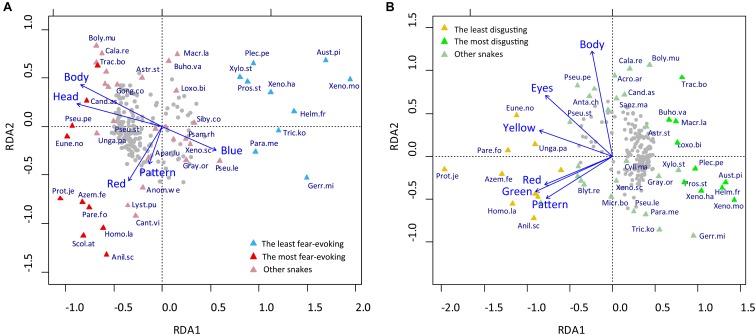
Redundancy Analysis (RDA) of the characters determining the ranks of fear and disgust elicited by snake stimuli used in Experiment 1. **(A)** Analysis of the fear ranks. The model explained 41.39% of the full variability, RDA1 = 0.2784% of variability and RDA2 = 0.0714% of variability. **(B)** Analysis of the disgust ranks. The full model explained 44.77% of the variability, RDA1 = 24.75% of variability, RDA2 = 10.76% of variability. For a better visualization of the fear/disgust ranks polarity, 10 species with the largest and lowest mean ranks of fear/disgust are mapped onto the pictures. For the description of the snake species abbreviations, see Supplementary Material 1.

We employed RDA to examine the contribution of various explanatory variables to the rankings of fear evoked by snakes. We used the automatic model-building feature based on both the Akaike criterion and permutation *P*-values. Both methods agreed on inclusion of the following variables into the reduced model, which were confirmed as significant by the sequential “Type I” test (*n* permutations = 10 000): head size (*F*_1,39_ = 12.095, *p* < 0.0001), red color (*F*_1,39_ = 4.778, *p* = 0.0029), pattern (*F*_1,39_ = 3.952, *p* = 0.0070), body size (*F*_1,39_ = 4.096, *p* = 0.0038), and blue color (*F*_1,39_ = 2.615, *p* = 0.0306). The RDA model has generated five constrained axes, which explained 41.39% of the full variability (Figure 2A).

The same procedure was used to find out which variables affect disgust rankings. The final model included the body size (*F*_1,38_ = 6.871, *p* = 0.0002), green color (*F*_1,38_ = 6.353, *p* = 0.0002), eyes size (*F*_1,38_ = 5.316, *p* = 0.0009), red color (*F*_1,38_ = 4.570, *p* = 0.0015), pattern (*F*_1,38_ = 4.549, *p* = 0.0019), and yellow color (*F*_1,38_ = 3.148, *p* = 0.0150). The six constrained axes explained 44.77% of the full variability (Figure 2B).

Scores of the first axes on the RDA visualization of fear (Figure 2A) and disgust (Figure 2B) rankings correspond to the mean rankings of the respective emotions; RDA1 scores x mean fear: *r*^2^ = 95,4%; RDA1 scores *x* mean disgust: *r*^2^ = -95,8%. The results show that similar characteristics affect the rankings of fear and disgust, but in the opposite directions. Big brightly red-colored snakes with an expressive head and complex scale pattern were ranked as the most fear-evoking, but the blue color present on some snakes in form of a light reflection caused them to be perceived as non-fearful. On the contrary, bright green, red, and yellow colors, large size, big eyes, and a complex pattern characterized snakes perceived as the least disgusting.

### Discussion

Most participants agreed on the position of snakes that were ranked as the most fear- or disgust-eliciting, and also on the snakes who are least fear- or disgust-eliciting. The reason behind this may be that the snakes “in the middle” of the scale may either evoke none of the studied emotions at all, or evoke both emotions at the same time, hence the disagreement. Contrastingly, the respondents agreed on the snakes placed at the extreme ends, which suggest that these snakes unambiguously either evoke the given emotion, or do not evoke it at all. The RDA analysis revealed the characteristics specific for snakes ranked as the most fear-eliciting – these included the large body size, bright red coloration, body shape with a distinctive head (as compared to the non-fear eliciting snakes with a small, almost indistinguishable head), and complex pattern. This pattern may include both contrasting ornaments and/or distinctive, raised scales, typical for some groups such as the vipers.

The results suggest that the same characteristics determine whether the snake will be rated as highly fear-evoking or disgusting, only in the opposite direction. This really applies to a number of characteristics: bulky, red, textured snakes are fear-evoking, while thin snakes with low red coloration and smooth texture are disgusting. This overlap of characteristics leads into a partial overlap (correlation) of the fear and disgust axes (*r*^2^ = 38.39%, *p* = 0.00001). However, each emotion can also be elicited by other specific characteristics, e.g., small eyes and dull coloration in the case of disgust-eliciting snakes. In Landová et al. (2018a), certain snake characteristics are associated with high fear rankings, including a short body length, wide head, and overall darkness. Moreover, the characteristics specific for disgust and fear elicitation are not mutually exclusive and can be present on one snake, e.g., the Northern Eyelash Boa (*Trachyboa boulengeri*) obtained high ranks of both fear and disgust. This snake has dull, brown coloration and a chunky body with sharp, distinctive scales, and represents a good example showing that when specific characteristics mix, the snake can evoke a combination of emotions.

Our findings concur with recent descriptions that European respondents fear the viper and viper-like snakes (Landová et al., 2018a), in which we showed that the evaluation of vipers as the most dangerous snakes is shared across people from Europe and Near East. As the only dangerous snakes in Europe (and adjacent Asian areas) are the vipers, these results may not be that surprising. However, we obtained similar results from studies in sub-Saharan Africa (Frynta, in preparation), where people also mainly fear vipers, even though there are deadly elapids present. One explanation can be that the vipers, being ambush predators, want to warn others of their presence and thus use specific characteristics, such as sharp edges and contrasting patterns, as aposematic signalization to deter enemies (Souchet and Aubret, 2016). Another explanation may be that the vipers are rather very cryptic and unseen (wanting to merge with the background to ambush a prey), but humans, being often unintended victims of encounters with vipers, perfected attentional mechanisms to effectively recognize and avoid these snakes. More research is needed to resolve this. Moreover, position of the snake plays role in human emotional response. Snakes in a striking posture with a coiled body and S-shaped contracted neck ready to attack are detected faster (Masataka et al., 2010) and trigger more intensive fear (Schaefer et al., 2014).

In most cases, the snakes ranked as the most disgusting were small, harmless species of blind snakes (Typhlopidae) living underground. As these species pose no danger to humans (both in terms of bite and possible contamination; O’Shea, 2018), we can assume that they evoke disgust only due to their close resemblance to phylogenetically unrelated, but morphologically very similar species (such as maggots or parasitic worms causing diseases; Muller and Wakelin, 2002). These animals share slimy look, pale pink coloration, little to no eyes and visually undefined (indistinguishable) head and tail, i.e., characteristics identified as disgust-evoking in this study.

## Experiment 2. Snakes Evoking Discrete Emotions

### Materials and Methods

#### Selection and Preparation of Stimuli

Picture sets used in this experiment consisted of 40 individual snakes for each discrete emotion (40 fear/40 disgust, further referred to as sets F and D). The sets F and D were printed and mixed into one large set consisting of 80 picture cards. They were constructed by identifying the subfamilies that were ranked as the most fear-evoking and disgust-evoking in Experiment 1 and selecting closely related subfamilies/species with similar morphology (see Supplementary Material 1). The subfamilies ranked as the most fear-evoking, i.e., Crotalinae, Viperinae, and Azemiopinae, all belonged to the family Viperidae. We selected morphologically defined species from these groups that corresponded to the results of Experiment 1: snakes with large body size, expressive scales with contrasting patterns, and bright coloration (Figure 1B).

The subfamilies Xenotyphlopinae, Typhlopinae, and Anomalepidinae of the Typhlopoidea group (blind snakes) were ranked as the most disgust-evoking. Snakes from these groups live usually underground and are rarely seen which made it difficult to complete a 40-picture set. To complete the set, we added species from the subfamilies Leptotyphlopinae (Typhlopoidea) and Chariniinae (Boidae) that morphologically correspond to the most disgusting snakes and their characteristics as analyzed in Experiment 1: species with thin bodies, smooth texture, small eyes, and dull coloration. We then searched for as many different pictures of each of the selected species/groups as possible on the Internet and modified them in the same way as in Experiment 1 (Figure 1C).

#### Testing Emotional Response to Snakes

The emotional response to snakes on these pictures was measured using the same method as in Experiment 1. However, unlike in Experiment 1, all respondents (*n* = 172, 118 women, 54 men; mean age = 25.23; *SD* = 9.43) sorted the set twice, once according to perceived fear and the other time according to disgust, while the order of sorting was balanced. The data about gender, age, and affiliation toward snakes of each respondent were collected, similarly as in Experiment 1. Additionally, we collected the results of the SNAQ (Polák et al., 2016) and DS-R questionnaires (Haidt et al., 1994; modified by Olatunji et al., 2007) from 155 respondents. However, due to a limited focus of this article, results concerning these data will be published in a separate article. The SNAQ score nevertheless highly correlated with the “affiliation toward snakes” variable (*r* = 0.6683; *p* < 0.0001). This simplified measure of self-assessed “snake fear” can thus be considered a suitable replacement for the needs of this article that is mainly focused on the variability among the stimuli.

The distribution and median of SNAQ and DS-R in our sample was comparable to the sample of respondents used in previous studies (SNAQ *n* = 594; DS-R *n* = 1006; Polák et al., 2016, 2018).

#### Explanatory Variables

Similarly to experiment 1, various data regarding the characteristics of snakes included in the large set were extracted. However, since their selection was mainly based on morphological similarity of the species with extreme rank values, we omitted the body length measurements and only analyzed color and pattern characteristics using Barvocuc: red < 350°; 18°), orange (corresponding to brown in all of the snakes) < 18°; 45°), yellow < 45°; 63°), green < 63°; 170°), blue < 170°; 270°), violet-rose < 270°; 350°), black (*L* < 0.25), white (*L* > 0.8), gray (*S* < 0.15), mean S, mean L, pattern, and opaque pixels. Transformation of the data was the same as in the Experiment 1.

#### Statistical Analysis

See Experiment 1. Additionally, for each respondent, we counted how many species from set F received a rank > 40. This value (further referred to as disagreement count) quantifies disagreement of the ranking performed by a particular respondent with grouping the snake stimuli into D or F. Moreover, the data were analyzed by a Cluster Analysis (CA). Manhattan distances (non-standardized) were selected as metrics and unweighted pair-group average UPGMA as a clustering method.

### Results

#### Agreement Among Respondents

In the case of F and D photo sets (ranked together as one large set), RDA analysis of the fear ranks included gender, age, affiliation, and order of the task. The full RDA model explained 3.16% of the full variability among the respondents. Sequential “Type I” ANOVA (*n* permutations = 10 000) confirmed that only the effect of affiliation was significant: affiliation: *F*_1,167_ = 2.056, *p* = 0.0062; gender: *F*_1,167_ = 1.139, *p* = 0.2784; age: *F*_1,167_ = 1.341, *p* = 0.1442; task order: *F*_1,167_ = 0.913, *p* = 0.5504. A reduced model, which included only the affiliation, explained 1.19% of the full variability (ANOVA: *F*_1,170_ = 2.051, *p* = 0.005). In case of the disgust ranks, the full model explained 3.89% of the full variability: affiliation: *F*_1,167_ = 2.7576, *p* = 0.0011; gender: *F*_1,167_ = 1.611, *p* = 0.0560; age: *F*_1,167_ = 1.262, *p* = 0.1948; task order: *F*_1,167_ = 1.132, *p* = 0.2798. Reduced model explained 1.58% variability; ANOVA affiliation: *F*_1,170_ = 2.7414, *p* = 0.0009. The data from both genders (separately for set D and F) were pooled for all further analysis.

Agreement among the respondents on the snake’s position within the particular set supports the distinctiveness of both emotions evoked by snakes from each set. When the respondents sorted the mixed sets according to evoked fear, the fear-evoking snakes were positioned among the first 40 places, while the rest (the disgust-evoking snakes) occupied the 41st to 80th place by 96 out of 172 respondents (55.8%). Additional 49 respondents showed only marginal disagreement (1–4 counts), thus 84.3% of respondents disagreed in the case of less than 10% of stimuli. Similar but opposite effect was found when sorting the sets according to evoked disgust, full-agreement in 80 (46.5%) and marginal disagreement in 53 out of 172 respondents (30.8%).

Consequently, when the respondents sorted the mixed sets according to evoked fear or disgust, Kendall’s Ws were high, 0.725 and 0.643, respectively. However, when we computed Kendall’s W for snakes from just one of the sets, the agreement was quite low; set F sorted by evoked fear: *W* = 0.076; set D sorted by evoked disgust *W* = 0.220. This means that within the F set, all stimuli were equally fear-evoking, and within the D set, all stimuli were equally disgust-evoking. Similarly, there was low agreement on disgust evoked by snakes within the F set (*W* = 0.113), as these snakes apparently do not evoke such emotion. In contrast, agreement on fear evoked by disgust-evoking snakes (set D) was exceptionally high (*W* = 0.503). We also expected a low *W* value in this case, but this high value suggests that there is still some variability among this group of stimuli. However, since all other analyses (PCA and CA) support exclusiveness of the disgust-evoking snakes, this agreement is presumably an artifact. Due to the respondents’ effort to solve a non-sensical task, they might have used a different guide when sorting the pictures.

#### Examination of Fear Rankings of Disgusting Snakes

To explore which characteristics affect fear rankings of the disgusting snakes within the D set, we performed a GLM analysis. The initial full model included all the explanatory variables as described within the section 3.1. After reduction using the Akaike Information Criterion (AIC; Akaike, 1998), the model explained as much as 90.93% of variation in the rankings (*p* < 0.0001). The white (*F*_1,32_ = 36.81, *p* < 0.0001), reddish brown (*F*_1,32_ = 44.31, *p* < 0.0001), and violet-rose color (*F*_1,32_ = 77.81, *p* < 0.0001) affected the fear rankings negatively, while orange brown (*F*_1,32_ = 129.39, *p* < 0.0001) and the opaque pixels (*F*_1,32_ = 107.38, *p* < 0.0001) affected them positively. The blue color and pattern stayed in the model but remained insignificant.

#### Categorization of Snakes Based on Fear and Disgust Evaluation

**FIGURE 3:**
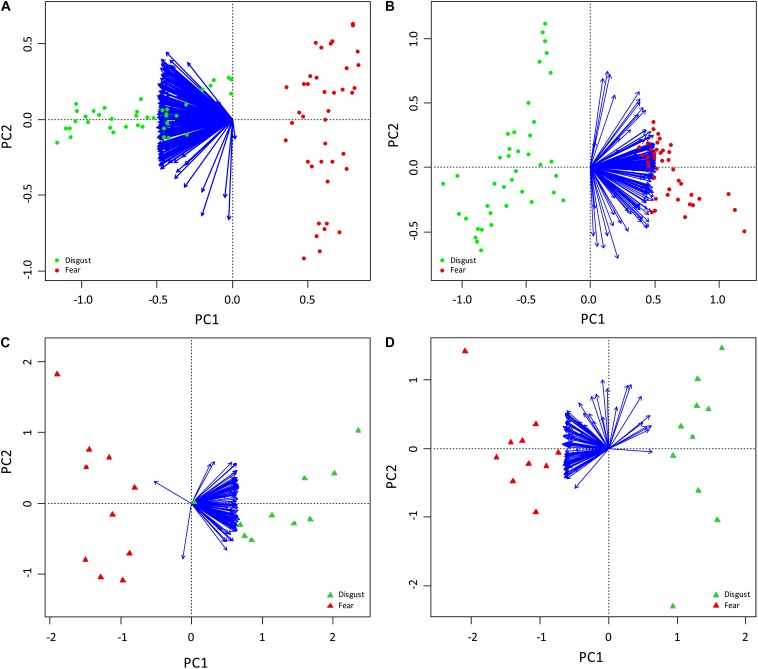
PC Analyses of the fear **(A,C)** and disgust **(B,D)** ranks in the Experiments 2 **(A,B)** and 3 **(C,D)**. The arrows correspond to the respondents, the dots and triangles represent the snakes. In all pictures, PC1, which explains 70.82, 64.89, 68.72, and 59.8% of the full variability in the **A–D** cases, respectively, clearly corresponds with the separation of the snakes from two pre-defined distinct categories, one consisting of only fear-eliciting snakes (shown in red) and other of only disgust-eliciting snakes (shown in green).

To further confirm distinctiveness of the snake groups, we performed a PCA based on the fear rankings, which generated 79 unconstrained axes. However, the eigenvalues of all but the first one (15.295) were lower than 1. The first principal component axis (PC1) accounted for 70.82% of variation in fear rankings and corresponded to the partition of snakes ranking as fear-evoking and “harmless” (evoking no fear; see Figure 3A). PCA based on the disgust rankings generated similar results. The analysis also resulted in 79 unconstrained axes, with eigenvalues higher than 1 in only the first two (14.0147 and 1.36652 for PC1 and PC2, respectively). In this case, the first axis accounting for 64.89% variation corresponded to the disgust-fear diversification of the two picture sets (Figure 3B). The second axis accounted only for 6.33% of variation in the disgust rankings. It is unknown which factor feeds this axis, but its effect is small compared to the effect of the set type (PC1).

*Post hoc* comparisons among the mean ranks of individual species revealed that all comparisons between fear-evoking and disgust-evoking stimuli were significant (see Supplementary Material 2b). This further confirms the distinctiveness of these categories of stimuli regarding the evaluation of both fear and disgust.

**FIGURE 4:**
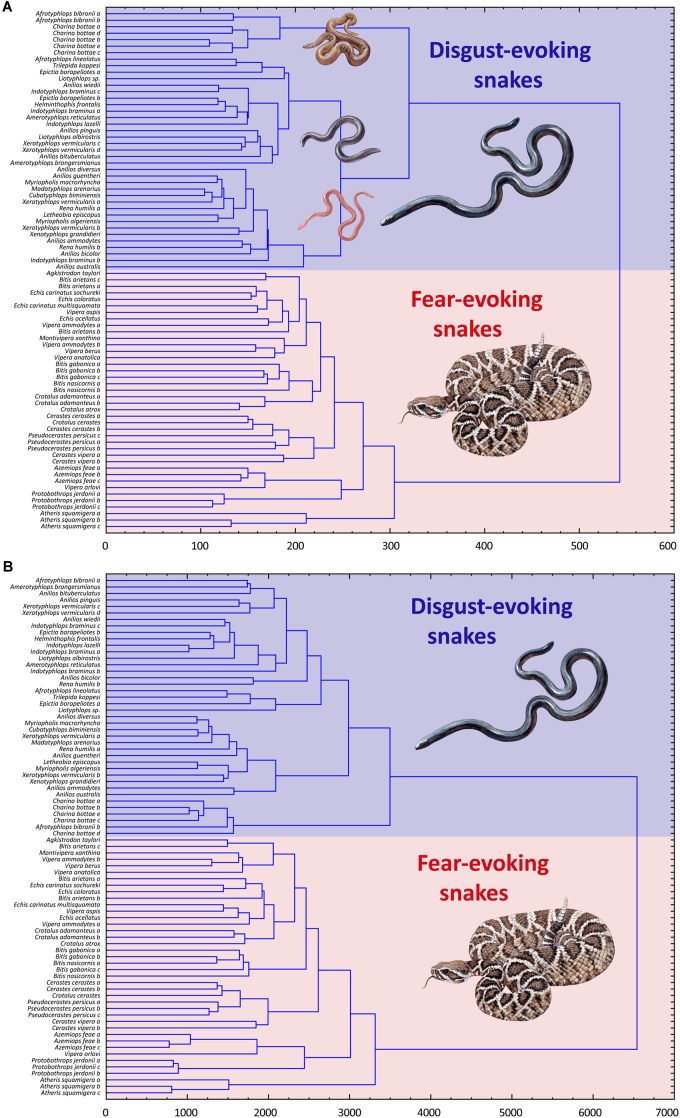
Cluster analysis (CA) of the fear **(A)** and disgust **(B)** ranks of the snakes from the Experiment 2. Both pictures show a separation of the snakes from the two pre-defined distinct categories, one consisting of only fear-eliciting snakes and other of only disgust-eliciting snakes. In case of the fear ranks **(A)**, the respondents also identified three different groupings of the disgust-eliciting snakes set. This, however, might be a result of a non-sensual task as there might be no actual variability in fear of snakes that evoke little to no fear.

Subsequent cluster analyses based on the (a) fear and (b) disgust rankings of all 80 snakes confirmed these results. Both trees confirmed strict distinctiveness of the F and D sets, regardless of the type of ranking (fear or disgust). Thus, when ranking the mixed F and D sets according to perceived fear, the respondents clearly divided the sets, ranking the F set as more fear-evoking than the D set. When ranking the sets according to the perceived disgust, the results were the same but opposite – the D set snakes were all rated as more disgusting than the F snakes (Figure 4A,B).

### Discussion

Based on the results of the first experiment, we created two groups of snakes, each consisting of snakes similar to those rated as the most fear- (F) or disgust-evoking (D). The respondents strongly agreed on the overall position of these snakes: they were able to cluster together snakes from each pre-defined group, sorting the D-snakes together as more disgusting and the F-snakes as more fear-evoking. This is noteworthy considering the fact that the variability in this new set has been considerably reduced compared to Experiment 1, making the task harder for the respondents. However, there was low to no agreement within the particular set. F-snakes evaluated according to fear and disgust were ranked in a random order, which means that there was no meaningful variability within the set in the perceived fear: all the snakes within the fear category evoked fear equally. A similar effect was observed when the participants evaluated disgusting snakes according to perceived disgust. Interestingly, the D set evaluated according to perceived fear was an exception, as the respondents fairly agreed on the relative snakes’ fearfulness (*W* = 0.503). The results of a subsequent analysis show that most of the variability (90.93%) can be explained by a few factors: large (bulky) brown snakes were evaluated as fear-evoking, while light, reddish-brown, and pink-purple snakes were evaluated as less fear-evoking.

A possible explanation of this observation can be that we failed to select the morphotypes exclusively evoking disgust. The CA (see Figure 4A) suggests a separation of the *Charina-A. bibronii* complex, i.e., brown bulky snakes. Bulky snakes were evaluated as fearful also in another study (in form of the wide head and tail, Landová et al., 2018a). Although this study suggests that this *Charina-A. bibronii* complex is equally disgusting as other snakes within the D set, we cannot rule out the possibility that they also evoke more or less fear. Another study based on the physiological measurement of the emotional response would be needed to resolve this. However, neither brown color nor bulkiness were the factors to affect the evaluated fear of the full snake set (in Experiment 1), which hints for another explanation of this observation.

Another explanation can be that the disgusting snakes do not evoke fear at all and thus the task to sort the stimuli according to perceived fear was non-sensual. Thus, the respondents either used another (unknown) scale (e.g., beauty/valence in general), or just categorized the snakes according to the residual morphological variability (i.e., bulkiness and color), which was their only lead [similarly, to the unsupervised categorization as described by Pothos and Chater (2002) or Pothos and Close (2008)]. The CA of the fear ranks of the D snakes show that the data form three main groups: the brown bulky snakes (*Charina-A. bibronii* complex), pink snakes, and dark snakes. The respondents agree on the order of these groups, i.e., the supposedly “most fearful” are the *Charina-A. bibronii*, then the dark snakes, and the least “fearful” are the pink snakes, but there is no agreement on the order within the sets (dark snakes *W* = 0.142; pink snakes *W* = 0.145). However, it is possible that the respondents do not really experience the fear emotion. Instead, they are able to estimate the degree of “fearfulness” based on these characteristics, which belong to the fear-inducing ones and which would really evoke fear in a different context or in the presence of other fear-inducing characteristics. The dark color (lightness) was responsible for the evaluation of snakes as more fear-inducing and pink as less fear-inducing in another study (Landová et al., 2018a). To resolve that, another study focused on this issue would be needed.

For future research of human responses to snakes, it is important to purify the image stimuli within each set targeted at triggering certain emotion, e.g., fear or disgust, to make sure that all the images within the given set elicit the desired emotional response only. This is especially crucial in studies monitoring continuous brain stimulation using functional neuroimaging methods.

## Experiment 3. Illustration of Snakes Evoking Discrete Emotions

### Materials and Methods

#### Selection and Preparation of Stimuli

The problem with photos found on the Internet is that each photo is taken under different conditions, is of a different quality, etc., and full standardization is nearly impossible. Additionally, copyright restrictions usually make it difficult to gather a full set of needed species for a wide use above the scope of a single experiment; especially if some species are rare and hardly ever photographed. Thus, our aim was to create a set of fully standardized illustrations to be available for further research. We randomly selected 20 species from sets D and F (10 from each) and commissioned painted illustrations of these species by a freelance artist. These photographs are publicly available for download at the Mendelay Data Repository (see section 8; 10 × 15 cm at 600 dpi, PNG picture files with transparent background).

#### Testing Emotional Response to Snakes

The set of 20 illustrated snakes was handled the same way as in Experiment 2: 104 respondents in total (78 women, 26 men; mean age = 33.93; *SD* = 14.85) sorted the snake illustrations according to both perceived fear and disgust. The order of both tasks was counter-balanced. The data about gender, age, and affiliation toward snakes of each respondent were collected similarly, as in Experiment 1.

#### Statistical Analysis

Similar to Experiment 1.

### Results

#### Agreement Among Respondents

**FIGURE 5:**
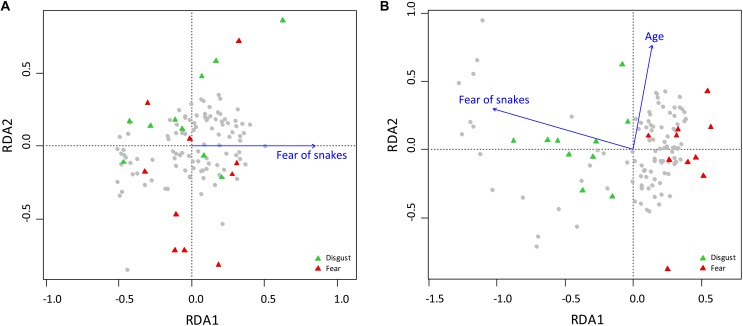
Redundancy Analysis of the characters determining the ranks of fear and disgust elicited by snake stimuli used in Experiment 3. **(A)** The model (RDA1) explains only 2.06% of the full variability, but it does not explain the separation of the disgust- and fear-eliciting snake categories. **(B)** Analysis of the disgust ranks. RDA1 explains 7.21% of the full variability and corresponds to the separation of the two snake groups. RDA2 (corresponding to age) explains 1.62% of the full variability, but does not contribute to the variability explaining the category separation.

RDA analysis of the fear ranks included gender, age, affiliation, and order of the task. The full RDA model explained 5.65% of the full variability among the respondents. Sequential “Type I” ANOVA confirmed that only the effect of affiliation was significant: affiliation: *F*_1,99_ = 2.161, *p* = 0.0089; gender: *F*_1,99_ = 1.148, *p* = 0.2982; age: *F*_1,99_ = 1.137, *p* = 0.2963; and task order: *F*_1,99_ = 1.481, *p* = 0.1056. A reduced model, which included only the affiliation, explained 2.06% of the full variability (ANOVA: *F*_1,102_ = 2.145, *p* = 0.0098). In case of the disgust ranks, the full model explained 11.26% of the full variability: affiliation: *F*_1,99_ = 7.097, *p* < 0.0001; gender: *F*_1,99_ = 1.396, *p* = 0.1807; age: *F*_1,99_ = 2.757, *p* = 0.0198; and task order: *F*_1,99_ = 1.308, *p* = 0.2149. Reduced model, which included affiliation and age, explained 8.83% variability: RDA1 = 7.21% (corresponding to the fear of snakes; *F*_1,101_ = 7.048, *p* < 0.0001) and RDA2 = 1.62% (age; *F*_1,101_ = 2.738, *p* < 0.0230; see Figure 5A,B).

A relationship between affiliation toward snakes and miscounts in fear and disgust categorization of snakes was positively correlated only in the case of disgust (Spearman *r* = 0.264, *p* = 0.0067), but not fear evaluation (Spearman *r* = 0.328, *p* = 0.0007).

**FIGURE 6:**
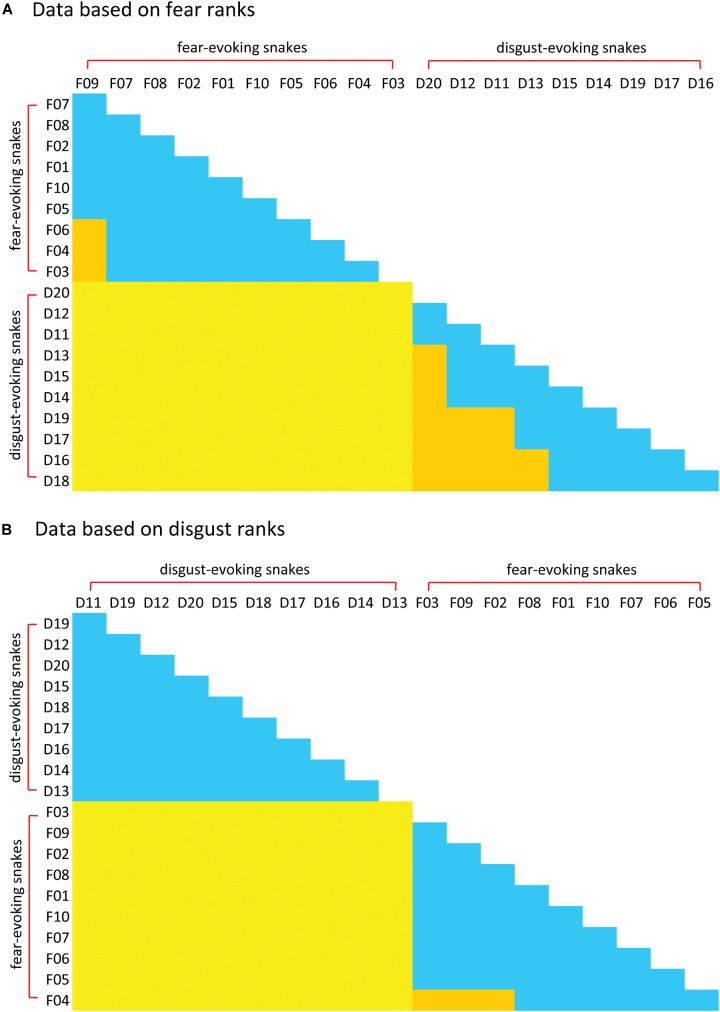
Matrix of *post hoc* Friedman-Neményi comparisons among the rankings of individual snake stimuli in Experiment 3. Species are arranged in an ascending order according to their mean rank of **(A)** fear and **(B)** disgust. Friedman test of the entire matrix was highly significant. Significant comparisons (*p* < 0.05) are marked yellow (comparisons between fear-evoking and disgust-evoking snakes) or orange (comparisons within groups) colors. Non-significant comparisons are denoted by blue. For mean ranks and *P*-values, see Supplementary Material 2.

The pattern of agreement on the position of disgust- vs fear-evoking snakes confirmed the results of Experiment 2. *Post hoc* comparisons among the mean ranks of the individual species revealed that all comparisons between the fear-evoking and disgust-evoking stimuli were significant (see Figure 6 and Supplementary Material 2c). When the respondents sorted the mixed sets according to fear, the fear-evoking snakes occupied the top 10 places, while the rest (i.e., the disgust-evoking snakes) occupied the 11st to 20th place by 61 out of 104 respondents (58.7%). Additional 27 (26%), 7 (6.7%), 4 (3.8%), and 3 (2.9%) respondents showed only marginal disagreement of one, two, three, and four miscounts (when fear-evoking snakes were placed among the disgust-evoking snakes), respectively. One respondent did seven and another one eight miscounts. Similar but opposite effect was found when sorting the sets according to evoked disgust, full-agreement in 71 (68.3%) and marginal disagreement in 19 out of 104 respondents (18.3%).

Similarly, agreement as expressed by the Kendall’s W also replicated the pattern seen in Experiment 2: for all illustrated snakes sorted by fear: *W* = 0.698; for disgust: *W* = 0.437, for fear-evoking snakes sorted by fear: *W* = 0.104; for disgust-evoking snakes sorted by fear: *W* = 0.433; for fear-evoking snakes sorted by disgust: *W* = 0.118; and for disgust-evoking snakes sorted by disgust: *W* = 0.025.

#### Categorization of Snakes Based on Fear and Disgust Evaluation

The PCA also firmly repeated the pattern seen in Experiment 2. The PCA based on the fear rankings generated 19 unconstrained axes, the eigenvalues of seven of which were higher than 1. The first axis (eigenvalue of 71.46) explained 68.72% of variability and corresponded to the partition of snakes ranking as fear-evoking and “harmless” (see Figure 3C). All other axes explained a very small portion of variability (5.13% and lower) when compared to the first axis. The PCA based on the disgust rankings generated similar results: 19 unconstrained axes with the eigenvalues of 62.18 and 10.46 PC1 for PC1 and PC2, respectively. Also in this case, the first axis accounting for 59.8% variation corresponded to the “very disgusting – not disgusting at all” diversification of the two picture sets. The second axis accounted for 10.06% variation in disgust rankings (Figure 3D).

**FIGURE 7:**
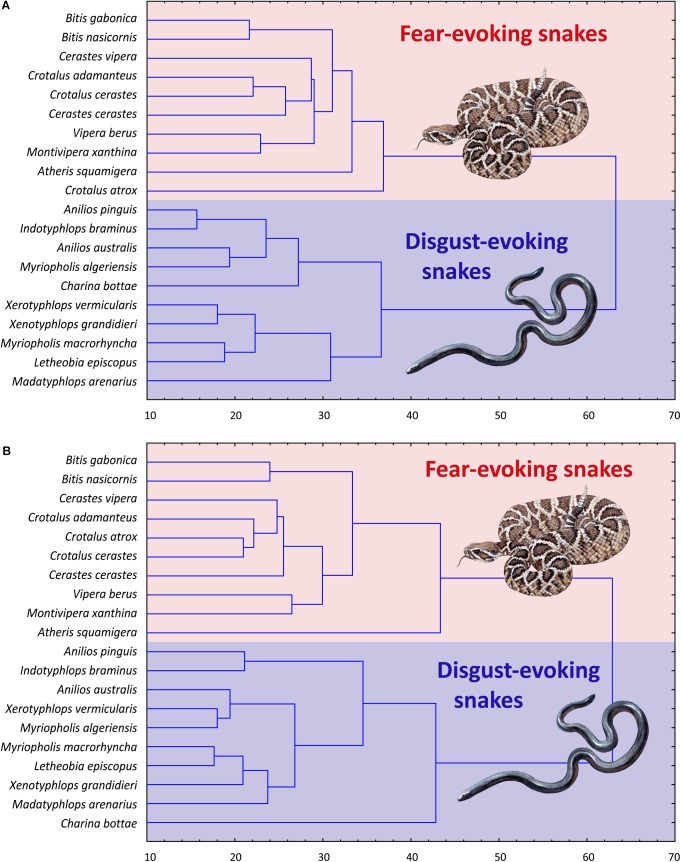
Cluster analysis of the fear **(A)** and disgust **(B)** ranks of the snakes from the Experiment 3. Both trees confirmed strict distinctiveness of the fear- and disgust-evoking clusters, regardless of the ranking type.

The subsequent CA based on the fear and disgust rankings of all the 20 snakes confirmed these results. Both trees confirmed strict distinctiveness of the fear- and disgust-evoking clusters, regardless of the ranking type (Figure 7).

### Discussion

The aim of the final experiment was to develop a standardized set of pictures that could be used in future research of snake phobia. Ideally, such stimuli should unambiguously evoke either fear or disgust of snakes. Therefore, to acquire comparable, standardized pictures available for use, we commissioned professionally painted illustrations of 20 selected species, ten from each category, and we re-tested their emotional valence to validate their potential to trigger a discrete emotional response.

Previous work has shown that photographs may be substituted for live snakes with no change in the quality and intensity of perceived emotion (Landová et al., 2012). However, we have demonstrated here that illustrations as well have fear- and disgust-evoking properties similar to photographs. This has been confirmed by various exploratory statistical approaches, all showing that the stimuli again formed two separate clusters (see Figure 3C,D, 5, 6). Although this categorization is highly significant, it is not absolute. A majority of the stimuli were categorized as expected following Experiment 2, however, (and similarly, to Experiment 2), the respondents sometimes misplaced a few species into the other category (e.g., *Charina bottae*). In case of disgust rankings, 12 respondents even inverted the scale, placing the snakes from the disgust category as less disgusting in average than the fear-eliciting ones. To examine the reason behind this observation, we decided to take a closer look on the characteristics of the respondents.

To minimize the effect on underlying snake phobias, we tested only healthy subjects (i.e., excluding clinically detected snake phobics). However, variability among the respondents in terms of snake-induced fear sensitivity and overall affiliation toward these animals was still present within the sample. Interestingly, even though this measurement of the subjective affiliation toward snakes was simple, it helped to explain the variability in the disgust rankings of the illustrated set that skewed the overall agreement when compared to the rankings of fear. The RDA analysis also revealed that this factor affected the structure of the disgust categorization into the two groups (Figure 5B). When compared to the pictures evaluation according to fear, the effect of the snake fear variable was small and did not contribute to the separation of the fear-disgust categories, although it was also revealed as significant (Figure 5A).

In summary, we found that a portion of the respondents with an overall negative affiliation toward snakes (probably high fear) rank the F-snakes as more disgusting than the D-snakes. This suggests that the illustrated F-snakes, which we consider fear-inducing, appear as much stronger negative stimuli to the respondents with high snake fear, regardless of the emotional categorization. This may also hint for a higher involvement of the disgust emotion (in addition to fear emotion) in the evaluation of snakes in people with strong negative affiliation toward snakes (and possibly in snake phobics). These findings are generally in line with current existing literature (Klieger and Siejak, 1997; Woody and Teachman, 2000; Smith and Davidson, 2006). Another explanation of this phenomenon might be that the respondents with high fear of snakes might perceive all snakes negatively, independently on their appearance. However, if this was the case, the categorization would be also distorted during the fear evaluation, which was not found.

## Limitations of the Study

This study was designed to analyze differences among stimuli, not among people. That is why it used the rank–ordering method instead of the Likert scale. This method is optimal for evaluating relative differences in mean ratings of the stimuli, but, on the other hand, it reduces the inter-respondents effect. Thus, it measures with high resolution the relative difference between stimuli, for example when we try to find out if the viper is perceived as more fearful than the grass snake. It is for this reason that the models incorporating the effect of sex, age, or affiliation to snakes can explain only a very little of the total variability (usually just 2–7%), because in fact they only evaluate differences in the relative order of the species.

Conversely, the absolute evaluation made on a Likert scale is more related to differences in experiencing emotions among respondents, e.g., when fear sensitive respondents attribute higher scores to both the viper and grass snake compared to less fearful subjects. This cannot be well inferred from our type of data, which is a limitation of the study. Nevertheless, we are fully aware of this issue and therefore, we have also collected the Likert scale data which will be analyzed in the next manuscript.

This might also explain the fact why at this time we did not give that much consideration to the questionnaires measuring fear and disgust sensitivity of the respondents and rather substituted the SNAQ and DS-R data by a single question of affiliation to snakes on a 7-point Likert scale. However, this only item highly correlates with the SNAQ (*r* = 0.6683; *p* < 0.0001), which we verified in Experiment 2.

## Conclusion

In this study, we show that “snakes” are not perceived as a single category. Based on their visual appearance, different snakes may evoke different emotions of fear and disgust, and these emotions are not necessarily mutually exclusive. Snakes can also be grouped into categories based on their morphotypes that evoke similar emotion(s). We were able to create sets of snakes that elicit almost exclusively disgust- and fear-response. Additionally, we do not only provide a smaller set of snake pictures with known emotional rankings, we also offer a “know-how” that will allow anyone else to include additional stimuli into each of the categories of “fear-evoking” and “disgust-evoking” snakes simply based on their unique characteristics.

When compared to existing affective picture databases, the variability among the snakes we present in this study is reduced to the actual variability among the snake species. It is thus especially useful in studies of the evolutionary basis of snake-induced emotional response that heeds the properties of snakes, their characteristics, and evolutionary history of the human-snake interaction. Moreover, we confirmed that snakes within the defined category (“fear-evoking” and “disgust-evoking”) form a homogenous group. Thus, these stimuli can be utilized in further studies of snake-induced fear in humans (incl. snake phobics) that require a larger number of homogenous pictures evoking discrete emotional response in a block-type design, such as fMRI and EEG.

In conclusion, snakes demonstrate an immense morphological variability with many patterns, and these patterns are associated with specific human reactions. The theoretical implication of this study is that humans do not react on a “snake” *per se* or some kind of its “snake essence,” but they can distinguish specific snake categories based on their morphotypes. For future research, it is critical that “snakes” cannot be considered as one homogenous category of stimuli.

## Data Availability

The datasets generated during and/or analyzed during the current study are available in the Mendeley repository, https://doi.org/10.17632/xktgm3j4s7.1.

## Ethics Statement

This study was carried out in accordance with the recommendations of Institutional Review Board (IRB), Faculty of Sciences, Charles University, approval n. 2013/7, and approval of the Ethical Committee of the National Institute of Mental Health n. 55/16, with the written informed consent from all subjects in accordance with the Declaration of Helsinki. The protocol was approved by the Institutional Review Board (IRB).

## Author Contributions

EL and DF contributed to the conception and designed the study. MJ, KS, DN, ŠP, and JP organized the database and performed the research. SR, DF, and MJ performed the statistical analysis. SR wrote the first draft of the manuscript. DF, EL, JP, and MJ wrote sections of the manuscript. Contributions of the first two authors (SR and MJ) are equal. All authors contributed to manuscript revision, read and approved the submitted version.

## Conflict of Interest Statement

The authors declare that the research was conducted in the absence of any commercial or financial relationships that could be construed as a potential conflict of interest.

## References

[B1] Akaike H. (1998). “Information theory and an extension of the maximum likelihood principle,” in *Selected Papers of Hirotugu Akaike*, eds Parzen E. Tanabe K. Kitagawa G. (New York, NY: Springer), 199–213. 10.1007/978-1-4612-1694-0_15

[B2] Angyal A. (1941). Disgust and related aversions. *J. Abnorm. Soc. Psychol.* 36 393–412. 10.1037/h0058254

[B3] Bar M. Neta M. (2006). Humans prefer curved visual objects. *Psychol. Sci.* 17 645–648. 10.1111/j.1467-9280.2006.01759.x 16913943

[B4] Bar M. Neta M. (2007). Visual elements of subjective preference modulate amygdala activation. *Neuropsychologia* 45 2191–2200. 10.1016/j.neuropsychologia.2007.03.008 17462678 PMC4024389

[B5] Barrett L. F. Lewis M. Haviland-Jones J. M. (eds) (2016). *Handbook of Emotions.* New York, NY: Guilford Publications.

[B6] Baynes-Rock M. (2017). Human perceptual and phobic biases for snakes: a review of the experimental evidence. *Anthrozoös* 30 5–18. 10.1080/08927936.2017.1270584

[B7] Biernacki P. Waldorf D. (1981). Snowball sampling: problems and techniques of chain referral sampling. *Sociol. Method Res.* 10 141–163. 10.1177/004912418101000205 26107223

[B8] Buck J. C. Weinstein S. B. Young H. S. (2018). Ecological and evolutionary consequences of parasite avoidance. *Trends Ecol. Evol.* 33 619–632. 10.1016/j.tree.2018.05.001 29807838

[B9] Chippaux J. P. (1998). Snake-bites: appraisal of the global situation. *Bull. World Health Organ.* 76 515–524. 9868843 PMC2305789

[B10] Cisler J. M. Olatunji B. O. Lohr J. M. (2009a). Disgust, fear, and the anxiety disorders: a critical review. *Clin. Psychol. Rev.* 29 34–46. 10.1016/j.cpr.2008.09.007 18977061 PMC2895912

[B11] Cisler J. M. Olatunji B. O. Lohr J. M. (2009b). Disgust sensitivity and emotion regulation potentiate the effect of disgust propensity on spider fear, blood-injection-injury fear, and contamination fear. *J. Behav. Ther. Exp. Psychol.* 40 219–229. 10.1016/j.jbtep.2008.10.002 19041963 PMC2895919

[B12] Curtis V. (2011). Why disgust matters. *Philos. Trans. R. Soc. B* 366 3478–3490. 10.1098/rstb.2011.0165 22042923 PMC3189359

[B13] Curtis V. Aunger R. Rabie T. (2004). Evidence that disgust evolved to protect from risk of disease. *Proc. Biol. Sci.* 271(Suppl. 4), S131–S133. 10.1098/rsbl.2003.0144 15252963 PMC1810028

[B14] Dan-Glauser E. S. Scherer K. R. (2011). The Geneva affective picture database (GAPED): a new 730-picture database focusing on valence and normative significance. *Behav. Res. Ther.* 43 468–477. 10.3758/s13428-011-0064-1 21431997

[B15] Davey G. C. (1994). Self-reported fears to common indigenous animals in an adult UK population: the role of disgust sensitivity. *Br. J. Psychol.* 85 541–554. 10.1111/j.2044-8295.1994.tb02540.x 7812671

[B16] de Jong P. J. Merckelbach H. (1998). Blood-injection-injury phobia and fear of spiders: domain specific individual differences in disgust sensitivity. *Pers. Individ. Differ.* 24 153–158. 10.1016/S0191-8869(97)00178-5 16274661

[B17] de Jong P. J. van Overveld M. Peters M. L. (2011). Sympathetic and parasympathetic responses to a core disgust video clip as a function of disgust propensity and disgust sensitivity. *Biol. Psychol.* 88 174–179. 10.1016/j.biopsycho.2011.07.009 21855601

[B18] de Pinho J. R. Grilo C. Boone R. B. Galvin K. A. Snodgrass J. G. (2014). Influence of aesthetic appreciation of wildlife species on attitudes towards their conservation in Kenyan agropastoralist communities. *PLoS One* 9:e88842. 10.1371/journal.pone.0088842 24551176 PMC3925186

[B19] Doctor R. M. Kahn A. P. Adamec C. (2010). *The Encyclopedia of Phobias, Fears, and Anxieties.* New York, NY: Infobase Publishing.

[B20] Ekman P. (1992). An argument for basic emotions. *Cogn. Emot.* 6 169–200. 10.1080/02699939208411068

[B21] Fox E. Griggs L. Mouchlianitis E. (2007). The detection of fear-relevant stimuli: are guns noticed as quickly as snakes? *Emotion* 7 691–696. 10.1037/1528-3542.7.4.691 18039035 PMC2757724

[B22] Frynta D. Marešová J. Øeháková-Petrù M. Šklíba J. Šumbera R. Krása A. (2011). Cross-cultural agreement in perception of animal beauty: boid snakes viewed by people from five continents. *Hum. Ecol.* 39 829–834. 10.1007/s10745-011-9447-2

[B23] Frynta D. Šimková O. Lišková S. Landová E. (2013). Mammalian collection on Noah’s ark: the effects of beauty, brain and body size. *PLoS One* 8:e63110. 10.1371/journal.pone.0063110 23690985 PMC3654911

[B24] Gilchrist P. T. Vrinceanu T. Béland S. Bacon S. L. Ditto B. (2016). Disgust stimuli reduce heart rate but do not contribute to vasovagal symptoms. *J. Behav. Ther. Exp. Psychol.* 51 116–122. 10.1016/j.jbtep.2016.01.005 26851836

[B25] Goodman L. A. (1961). Snowball sampling. *Ann. Math. Stat.* 32 148–170.

[B26] Grant S. Aitchison T. Henderson E. Christie J. Zare S. Mc Murray J. (1999). A comparison of the reproducibility and the sensitivity to change of visual analogue scales, Borg scales, and Likert scales in normal subjects during submaximal exercise. *Chest* 116 1208–1217. 10.1378/chest.116.5.1208 10559077

[B27] Grine F. E. Fleagle J. G. Leakey R. E. (eds) (2009). *The First Humans: Origin and Early Evolution of the Genus Homo.* Dordrecht: Springer, 10.1007/978-1-4020-9980-9

[B28] Guthrie G. Wiener M. (1966). Subliminal perception or perception of partial cue with pictorial stimuli. *J. Pers. Soc. Psychol.* 3 619–628. 10.1037/h0023197 5938997

[B29] Haberkamp A. Glombiewski J. A. Schmidt F. Barke A. (2017). The DIsgust-RelaTed-Images (DIRTI) database: validation of a novel standardized set of disgust pictures. *Behav. Res. Ther.* 89 86–94. 10.1016/j.brat.2016.11.010 27914317

[B30] Haidt J. McCauley C. Rozin P. (1994). Individual differences in sensitivity to disgust: a scale sampling seven domains of disgust elicitors. *Pers. Individ. Differ.* 16 701–713. 10.1016/0191-8869(94)90212-7

[B31] Isbell L. A. (2006). Snakes as agents of evolutionary change in primate brains. *J. Hum. Evol.* 51 1–35. 10.1016/j.jhevol.2005.12.012 16545427

[B32] Isbell L. A. Etting S. F. (2017). Scales drive detection, attention, and memory of snakes in wild vervet monkeys (*Chlorocebus pygerythrus*). *Primates* 58 121–129. 10.1007/s10329-016-0562-y 27517268

[B33] Kasturiratne A. Wickremasinghe A. R. de Silva N. Gunawardena N. K. Pathmeswaran A. Premaratna R. (2008). The global burden of snakebite: a literature analysis and modelling based on regional estimates of envenoming and deaths. *PLoS Med.* 5:e218. 10.1371/journal.pmed.0050218 18986210 PMC2577696

[B34] Kimball S. Mattis P. (1995-2005). *GIMP 2.8.16^E^, GNU Image Manipulation Software*^∗∗^. Available at: https://www.gimp.org/^∗^ 10.1371/journal.pmed.0050218 18986210 PMC2577696

[B35] Klieger D. M. Siejak K. K. (1997). Disgust as the source of false positive effects in the measurement of ophidiophobia. *J. Psychol.* 131 371–382. 10.1080/00223989709603523 9190054

[B36] Landová E. Bakhshaliyeva N. Janovcová M. Peléšková Š Suleymanova M. Polák J. (2018a). Association between fear and beauty evaluation of snakes: cross-cultural findings. *Front. Psychol.* 9:333. 10.3389/fpsyg.2018.00333 29615942 PMC5865084

[B37] Landová E. Poláková P. Rádlová S. Janovcová M. Bobek M. Frynta D. (2018b). Beauty ranking of mammalian species kept in the Prague Zoo: does beauty of animals increase the respondents’ willingness to protect them? *Sci. Nat.* 105:69. 10.1007/s00114-018-1596-3 30488357

[B38] Landová E. Marešová J. Šimková O. Cikánová V. Frynta D. (2012). Human responses to live snakes and their photographs: evaluation of beauty and fear of the king snakes. *J. Environ. Psychol.* 32 69–77. 10.1016/j.jenvp.2011.10.005

[B39] Lang P. Ohman A. Vaitl D. (1988). *The International Affective Picture System.* Gainesville, FL: University of Florida.

[B40] Libkuman T. M. Otani H. Kern R. Viger S. G. Novak N. (2007). Multidimensional normative ratings for the international affective picture system. *Behav. Res. Methods* 39 326–334. 10.3758/BF03193164 17695361

[B41] Likert R. (1932). A technique for the measurement of attitudes. *Arch. Psychol.* 22 1–55.

[B42] Lišková S. Frynta D. (2013). What determines bird beauty in human eyes? *Anthrozoös* 26 27–41. 10.2752/175303713X13534238631399

[B43] LiškováS.LandováE.FryntaD. (2015). Human preferences for colorful birds: vivid colors or pattern? *Evol. Psychol.* 13 339–359. 10.1177/14747049150130020325920889

[B44] LoBue V. DeLoache J. S. (2008). Detecting the snake in the grass: attention to fear-relevant stimuli by adults and young children. *Psychol. Sci.* 19 284–289. 10.1111/j.1467-9280.2008.02081.x 18315802

[B45] LoBue V. Deloache J. S. (2011). What’s so special about slithering serpents? Children and adults rapidly detect snakes based on their simple features. *Vis. Cogn.* 19 129–143. 10.1080/13506285.2010.522216

[B46] Marchewka A. Żurawski Ł. Jednoróg K. Grabowska A. (2014). The nencki affective picture system (NAPS): introduction to a novel, standardized, wide-range, high-quality, realistic picture database. *Behav. Res. Ther.* 46 596–610. 10.3758/s13428-013-0379-1 23996831 PMC4030128

[B47] Marešová J. Krása A. Frynta D. (2009a). We all appreciate the same animals: cross-cultural comparison of human aesthetic preferences for snake species in Papua New Guinea and Europe. *Ethology* 115 297–300. 10.1111/j.1439-0310.2009.01620.x

[B48] Marešová J. Landová E. Frynta D. (2009b). What makes some species of milk snakes more attractive to humans than others? *Theor. Biosci.* 128 227–235. 10.1007/s12064-009-0075-y 19890672

[B49] Masataka N. Hayakawa S. Kawai N. (2010). Human young children as well as adults demonstrate ‘superior’ rapid snake detection when typical striking posture is displayed by the snake. *PLoS One* 5:e15122. 10.1371/journal.pone.0015122 21152050 PMC2994910

[B50] Meno W. Coss R. G. Perry S. (2013). Development of snake-directed antipredator behavior by wild white-faced capuchin monkeys: I. Snake-species discrimination. *Am. J. Primatol.* 75 281–291. 10.1002/ajp.22106 23229464

[B51] Michałowski J. M. Droździel D. Matuszewski J. Koziejowski W. Jednoróg K. Marchewka A. (2017). The set of fear inducing pictures (SFIP): development and validation in fearful and nonfearful individuals. *Behav. Res. Methods* 49 1407–1419. 10.3758/s13428-016-0797-y 27613018 PMC5541104

[B52] Mikels J. A. Fredrickson B. L. Larkin G. R. Lindberg C. M. Maglio S. J. Reuter-Lorenz P. A. (2005). Emotional category data on images from the international affective picture system. *Behav. Res. Methods* 37 626–630. 10.3758/BF03192732 16629294 PMC1808555

[B53] Muller R. Wakelin D. (2002). *Worms and Human Disease.* New York, NY: CABI Publishing.

[B54] Muris P. Mayer B. Huijding J. Konings T. (2008). A dirty animal is a scary animal! Effects of disgust-related information on fear beliefs in children. *Behav. Res. Ther.* 46 137–144. 10.1016/j.brat.2007.09.005 17959140

[B55] Näsänen R. Ojanpää H. Kojo I. (2001). Effect of stimulus contrast on performance and eye movements in visual search. *Vis. Res.* 41 1817–1824. 10.1016/S0042-6989(01)00056-6 11369045

[B56] Öhman A. Flykt A. Esteves F. (2001). Emotion drives attention: detecting the snake in the grass. *J. Exp. Psychol. Gen.* 130 466–478. 10.1037/0096-3445.130.3.466 11561921

[B57] Öhman A. Mineka S. (2001). Fears, phobias, and preparedness: toward an evolved module of fear and fear learning. *Psychol. Rev.* 108 483–522. 10.1037/0033-295X.108.3.483 11488376

[B58] Öhman A. Mineka S. (2003). The malicious serpent: snakes as a prototypical stimulus for an evolved module of fear. *Curr. Dir. Psychol. Sci.* 12 5–9. 10.1111/1467-8721.01211

[B59] Öhman A. Soares J. J. (1994). “Unconscious anxiety”: phobic responses to masked stimuli. *J. Abnorm. Psychol.* 103 231–240. 10.1037/0021-843X.103.2.231 8040492

[B60] Öhman A. Soares S. C. Juth P. Lindström B. Esteves F. (2012). Evolutionary derived modulations of attention to two common fear stimuli: serpents and hostile humans. *J. Cogn. Psychol.* 24 17–32. 10.1080/20445911.2011.629603

[B61] Oksanen J. Blanchet F. G. Friendly M. Kindt R. Legendre P. McGlinn D. (2017). *Vegan: Community Ecology Package. R Package Version 2.4–5.*

[B62] Olatunji B. O. Huijding J. de Jong P. J. Smits J. A. (2011). The relative contributions of fear and disgust reductions to improvements in spider phobia following exposure-based treatment. *J. Behav. Ther. Exp. Psychol.* 42 117–121. 10.1016/j.jbtep.2010.07.007 20732677

[B63] Olatunji B. O. Williams N. L. Tolin D. F. Sawchuck C. N. Abramowitz J. S. Lohr J. M. (2007). The disgust scale: item analysis, factor structure, and suggestions for refinement. *Psychol. Assess.* 19 281–297. 10.1037/1040-3590.19.3.281 17845120

[B64] O’Shea M. (2018). *The Book of Snakes: A life-Size Guide to Six Hundred Species From Around the World.* Chicago, IL: University of Chicago Press.

[B65] Polák J. Landová E. Frynta D. (2018). Undisguised disgust: a psychometric evaluation of a disgust propensity measure. *Curr. Psychol.* 1–10. 10.1007/s1214

[B66] Polák J. Sedláèková K. Nácar D. Landová E. Frynta D. (2016). Fear the serpent: a psychometric study of snake phobia. *Psychiatr. Res.* 242 163–168. 10.1016/j.psychres.2016.05.024 27280527

[B67] Pothos E. M. Chater N. (2002). A simplicity principle in unsupervised human categorization. *Cogn. Sci.* 26 303–343. 10.1016/S0364-0213(02)00064-2

[B68] Pothos E. M. Close J. (2008). One or two dimensions in spontaneous classification: a simplicity approach. *Cognition* 107 581–602. 10.1016/j.cognition.2007.11.007 18083157

[B69] Prokop P. Fančovièová J. (2013). Does colour matter? The influence of animal warning colouration in human emotions and willingness to protect them. *Anim. Conserv.* 16 458–466. 10.1111/acv.12014

[B70] Prokop P. Fančovičová J. Kuèerová A. (2018). Aposematic colouration does not explain fear of snakes in humans. *J. Ethol.* 36 35–41. 10.1007/s10164-017-0533-9

[B71] Prokop P. Medina-Jerez W. Coleman J. Fančovièová J. Özel M. Fedor P. (2016). Tolerance of frogs among high school students: influences of disgust and culture. *EURASIA J. Math. Sci. Tech.* 12 1499–1505. 10.12973/eurasia.2016.1241a

[B72] Ptáčková J. Landová E. Lišková S. Kubìna A. Frynta D. (2017). Are the aesthetic preferences towards snake species already formed in pre-school aged children? *Eur. J. Dev. Psychol.* 14 16–31. 10.1080/17405629.2016.1144507

[B73] Pyron R. A. Burbrink F. T. Wiens J. J. (2013). A phylogeny and revised classification of Squamata, including 4161 species of lizards and snakes. *BMC Evol. Biol.* 13:93. 10.1186/1471-2148-13-93 23627680 PMC3682911

[B74] R Development Core Team (2010). *R: A Language and Environment for Statistical Computing.* Vienna: R Foundation for Statistical Computing.

[B75] Rádlová S. Landová E. Frynta D. (2018). Judging others by your own standards: attractiveness of primate faces as seen by human respondents. *Front. Psychol.* 9:2439. 10.3389/fpsyg.2018.02439 30618913 PMC6297365

[B76] Rádlová S. Viktorin P. Frynta D. (2016). *Barvocuc 2.0, Software for Color Image Analysis.* Bethesda: National Institute of Mental Health.

[B77] Rasband W. S. (1997–2008). *ImageJ.* Bethesda: National Institutes of Health.

[B78] Riegel M. Żurawski Ł. Wierzba M. Moslehi A. Klocek Ł. Horvat M. (2016). Characterization of the nencki affective picture system by discrete emotional categories (NAPS BE). *Behav. Res. Ther.* 48 600–612. 10.3758/s13428-015-0620-1 26205422 PMC4891391

[B79] Rozin P. Haidt J. McCauley C. R. (1999). “Disgust: the body and soul emotion,” in *Handbook of Cognition and Emotion*, eds Dalgleish T. Power M. J. (New York, NY: John Wiley & Sons Ltd), 429–445. 10.1002/0470013494.ch21

[B80] Sawchuk C. N. Lohr J. M. Tolin D. F. Lee T. C. Kleinknecht R. A. (2000). Disgust sensitivity and contamination fears in spider and blood–injection–injury phobias. *Behav. Res. Ther.* 38 753–762. 10.1016/S0005-7967(99)00093-5 10937424

[B81] Schaefer H. S. Larson C. L. Davidson R. J. Coan J. A. (2014). Brain, body, and cognition: neural, physiological and self-report correlates of phobic and normative fear. *Biol. Psychol.* 98 59–69. 10.1016/j.biopsycho.2013.12.011 24561099 PMC4251669

[B82] Schindler I. Hosoya G. Menninghaus W. Beermann U. Wagner V. Eid M. (2017). Measuring aesthetic emotions: a review of the literature and a new assessment tool. *PLoS One* 12:e0178899. 10.1371/journal.pone.0178899 28582467 PMC5459466

[B83] Silvia P. J. Barona C. M. (2009). Do people prefer curved objects? Angularity, expertise, and aesthetic preference. *Empir. Stud. Arts* 27 25–42. 10.2190/EM.27.1.b

[B84] Smith M. Davidson J. (2006). It makes my skin crawl.’: the embodiment of disgust in phobias of ‘Nature. *Body Soc.* 12 43–67. 10.1177/1357034X06061195

[B85] Soares S. C. Esteves F. Flykt A. (2009a). Fear, but not fear-relevance, modulates reaction times in visual search with animal distractors. *J. Anxiety Disord.* 23 136–144. 10.1016/j.janxdis.2008.05.002 18565724

[B86] Soares S. C. Esteves F. Lundqvist D. Öhman A. (2009b). Some animal specific fears are more specific than others: evidence from attention and emotion measures. *Behav. Res. Ther.* 47 1032–1042. 10.1016/j.brat.2009.07.022 19695561

[B87] Soares S. C. Lindström B. Esteves F. Öhman A. (2014). The hidden snake in the grass: superior detection of snakes in challenging attentional conditions. *PLoS One* 9:e114724. 10.1371/journal.pone.0114724 25493937 PMC4262429

[B88] Sobel I. (1978). Neighborhood coding of binary images for fast contour following and general binary array processing. *Comput. Vis. Graph* 8 127–135. 10.1016/S0146-664X(78)80020-3

[B89] Souchet J. Aubret F. (2016). Revisiting the fear of snakes in children: the role of aposematic signalling. *Sci. Rep.* 6:37619. 10.1038/srep37619 27886218 PMC5122844

[B90] Stark R. Walter B. Schienle A. Vaitl D. (2005). Psychophysiological correlates of disgust and disgust sensitivity. *J. Psychophysiol.* 19 50–60. 10.1027/0269-8803.19.1.50

[B91] StatSoft Inc. (2010). *Statistica (Data Analysis Software System), Version 9.1.* Tulsa, OK: StatSoft Inc.

[B92] Tomažič I. (2011). Seventh graders’ direct experience with, and feelings toward, amphibians and some other nonhuman animals. *Soc. Anim.* 19 225–247. 10.1163/156853011X578901

[B93] Trape J. F. Pison G. Guyavarch E. Mane Y. (2001). High mortality from snakebite in south-eastern Senegal. *Trans. R. Soc. Trop. Med. Hyg.* 95 420–423. 10.1016/S0035-9203(01)90202-0 11579888

[B94] Uetz P. Hošek J. (2018). *The Reptile Database.* Available at: http://www.reptile-database.org (accessed November 17, 2015).

[B95] Üher J. (1991). On zigzag desings: three levels of meaning. *Curr. Anthropol.* 32 437–439. 10.1086/203979

[B96] Valenta J. (2008). *Jedovatí Hadi. Intoxikace, Terapie.* Praha: Galén Press.

[B97] Van Le Q. Isbell L. A. Matsumoto J. Nguyen M. Hori E. Maior R. S. (2013). Pulvinar neurons reveal neurobiological evidence of past selection for rapid detection of snakes. *Proc. Natl. Acad. Sci. U.S.A.* 110 19000–19005. 10.1073/pnas.1312648110 24167268 PMC3839741

[B98] van Strien J. W. Eijlers R. Franken I. H. A. Huijding J. (2014). Snake pictures draw more early attention than spider pictures in non-phobic women: evidence from event-related brain potentials. *Biol. Psychol.* 96 150–157. 10.1016/j.biopsycho.2013.12.014 24374241

[B99] van Strien J. W. Isbell L. A. (2017). Snake scales, partial exposure, and the snake detection theory: a human event-related potentials study. *Sci. Rep.* 7:46331. 10.1038/srep46331 28387376 PMC5384215

[B100] Woody S. R. McLean C. Klassen T. (2005). Disgust as a motivator of avoidance of spiders. *J. Anxiety Disord.* 19 461–475. 10.1016/j.janxdis.2004.04.002 15721575

[B101] Woody S. R. Teachman B. A. (2000). Intersection of disgust and fear: normative and pathological views. *Clin. Psychol. Sci. Pract.* 7 291–311. 10.1093/clipsy.7.3.291

[B102] Wuensch K. L. (2015). *What is a Likert Scale? and How Do You Pronounce’ Likert?.* Greenville, NC: East Carolina University.

[B103] Zsido A. N. Arato N. Inhof O. Janszky J. Darnai G. (2018a). Short versions of two specific phobia measures: the snake and the spider questionnaires. *J. Anxiety Disord.* 54 11–16. 10.1016/j.janxdis.2017.12.002 29306023

[B104] Zsido A. N. Deak A. Bernath L. (2018b). Is a snake scarier than a gun? The ontogenetic–phylogenetic dispute from a new perspective: the role of arousal. *Emotion* [Epub ahead of print]. 30188154 10.1037/emo0000478

